# Transcriptome Analysis Reveals Differentially Expressed circRNAs Associated with Fecundity in Small-Tail Han Sheep Thyroid with Different FecB Genotypes

**DOI:** 10.3390/ani14010105

**Published:** 2023-12-27

**Authors:** Cheng Chang, Xiaoyun He, Ran Di, Xiangyu Wang, Miaoceng Han, Chen Liang, Mingxing Chu

**Affiliations:** 1 State Key Laboratory of Animal Biotech Breeding, Institute of Animal Science, Chinese Academy of Agricultural Sciences (CAAS), Beijing 100193, China; changcheng20200911@163.com (C.C.); hexiaoyun@caas.cn (X.H.); diran@caas.cn (R.D.); wangxiangyu@caas.cn (X.W.); 2 College of Animal Science, Shanxi Agricultural University, Taigu 030801, China; hanmiaoceng@163.com

**Keywords:** STH sheep, follicular phase, luteal phase, thyroid gland, litter size, CircRNA

## Abstract

**Simple Summary:**

The lambing trait is an important production trait in sheep, and the *FecB* gene is one of the major genes affecting sheep reproduction. In order to explore the molecular regulatory mechanisms affecting lambing number in sheep, we used RNA-seq technology to detect the thyroid transcircRNA recombinome expression patterns during the luteal and luteal phases in different *FecB* genotypes of small-tail Han sheep (STH), identified reproduction-related circRNAs in the thyroid gland of STH sheep, and performed bioinformatic analysis, RT-qPCR validation of these genes, and dual luciferase reporter gene experimental validation to predict their potential functions in repopulation and better reveal the molecular mechanisms of thyroid organization during reproduction in sheep.

**Abstract:**

Litter size is an economically important trait in sheep, and it is a complex trait controlled by multiple genes in multiple organs. Among them, the regulation of lamb number trait by the thyroid gland is a very important part. However, the molecular mechanisms of the thyroid gland in sheep reproduction remain unclear. Here, RNA-seq was used to detect transcriptome expression patterns in the thyroid gland between follicular phase (FP) and luteal phase (LP) in *FecB BB* (MM) and *FecB* ++ (ww) STH sheep, respectively, and to identify differentially expressed circRNAs (DECs) associated with reproduction. Bioinformatic analysis of the source genes of these DECs revealed that they can be enriched in multiple signaling pathways involved in the reproductive process of animals. We found that the source genes of these DECs, such as *GNAQ*, *VEGFC*, *MAPK1*, *STAT1,* and *HSD17B7,* may play important roles in the reproductive process of animals. To better understand the function of these DECs, we constructed circRNA–miRNA co-expression networks. Dual luciferase reporter assays suggested that a ceRNA regulatory mechanism between circ_0003259-oar-miR-133-*TXLNA* and circ_0012128-oar-miR-370-3p-*FGFR1* may hold. All of these DEC expression profiles in the thyroid gland provide a novel resource for elucidating the regulatory mechanisms underlying STH sheep prolificacy.

## 1. Introduction

The STH sheep is an important domesticated animal with the advantages of short generational intervals and stable genetic performance, which can provide humans with high-quality meat, wool, and other resources and play an important role in the livestock industry [1]. Lambing traits are important production traits in STH sheep and are affected by many factors, such as age, breed, and genetic factors. Among these factors, genetic elements are intrinsic and regulated by micro-effective polygenes [2]. Many genes have been identified that play an important role in sheep reproduction, among which *FecB* is the major gene, which is derived from a mutation at base 746 of the *BMPR1B* gene, also known as the *FecB* mutation [3]. The *FecB* gene has been shown to have a cumulative effect on the ovulation rate. Our previous study found that ewes carrying two *FecB* mutations (*FecB ^BB^*) had an average of three more lambs than ewes with the *FecB^++^* genotype [4]. This suggests a great potential for using the *FecB* mutation to breed multiparous meat sheep breeds.

The thyroid gland can synthesize and secrete TH, which consists mainly of T3 and T4. T4 is transported to various tissues throughout the body by the action of thyroid hormone transporter proteins, and deiodinase is present in some tissue cells and in the periphery to convert T4 to T3, which will bind to the thyroid hormone receptor with 10 times the affinity of T4. Thyroid hormones have been shown to affect the reproductive process in animals mainly by influencing the secretion of GnRH [5]. The mechanism is that secreted T3 acts on the KISS promoter (KISS) and RF-amino acid-related peptide (RFRP) in the hypothalamus to promote the release of GnRH from gonadotropin-releasing hormone (GnRH) neurons, which acts on the pituitary portal system. Promotes the synthesis and secretion of follicle-stimulating hormone (FSH) and luteinizing hormone (LH) by gonadal cells, which results in the regulation of mammalian reproductive processes [6,7]. Hypothyroidism has been found to delay the onset of puberty [8], reduce estrogen levels, and decrease endometrial tolerance in rats [9].

circRNAs are a class of endogenous non-coding RNA molecules with a closed-loop structure that regulate gene expression in eukaryotic cells mainly through the adsorption of miRNAs. With the development of high-throughput RNA sequencing technology, thousands of circRNAs have been identified, and more and more studies have shown that circRNAs are involved in the reproduction process of animals [10]. Liu et al. [11] conducted RNA-seq sequencing on ovarian tissues collected from single and multiparous Hanper sheep during the follicular and luteal phases, respectively. They identified several differentially expressed circRNAs, annotated these circRNAs for functional enrichment, and observed that these differentially expressed circRNAs were enriched in signaling pathways known to influence the reproductive processes in animals, such as EGF-EGFR-RAS-JNK, TGF-β, and thyroid hormone signaling pathways. Zhang et al. [12] identified several differential circRNAs by RNA-seq of porcine oocyte nuclei and found that circARMC4 affects chromosome arrangement, first polar body extrusion and early embryonic development in porcine oocytes. In our laboratory, several circRNAs associated with sheep reproduction were identified by RNA-seq analysis of hypothalamic tissues from STH sheep [13] and oviductal tissues from Yunshang black goats [14] at different reproductive cycles. In addition, we performed RNA-seq analysis of the thyroid gland of sheep with different *FecB* genotypes STH in the follicular phase, which resulted in the identification of several differentially expressed lncRNAs and mRNAs that can be enriched in signaling pathways associated with reproduction in sheep [15].

This study identified differentially expressed circRNAs and predicted their potential functions related to reproduction by transcriptomic analysis of the thyroid gland of STH sheep with luteal phase and follicular phase *FecB BB* and *FecB* ++ genotypes. This was designed to provide a new resource for better understanding the molecular mechanisms of circRNA regulation of reproduction in sheep.

## 2. Materials and Methods

### 2.1. Animals and Sample Collection

The *FecB* genotype was identified using TaqMan probes from 890 healthy non-pregnant sheep aged 2–4 years from a core breeding herd in the Luxi region of Shandong, China, selected by jugular vein blood collection. Twelve sheep were selected for the experiment with no significant differences in age, weight, length, or chest circumference [16]. All test ewes were treated for simultaneous estrus, and CIDR suppositories (300 mg progesterone, InterAg, New Zealand) were placed in the vagina of the ewes for 12 days and then withdrawn. Fifty hours after bolus withdrawal, three *FecB BB* genotype sheep (MM) and three *FecB* ^++^ genotype sheep (ww) were euthanized (intravenous pentobarbital 100 mg/kg) and thyroid tissue was collected; 7 d after bolus withdrawal, three additional MM and three ww ewes were euthanized and thyroid tissue collected. The collected samples were temporarily stored in liquid nitrogen and brought back to the laboratory for immediate storage in a −80 °C refrigerator for later experiments. The collected thyroid tissues were divided into four groups: 3 *FecB* BB genotype sheep thyroid tissues of the follicular stage (MM-FT), 3 *FecB BB* genotype sheep thyroid tissues of the luteal stage (MM-LT), 3 *FecB* ^++^ genotype sheep thyroid tissues of the follicular stage (ww-FT), and 3 *FecB* ^++^ genotype sheep thyroid tissues of the luteal stage (ww-LT). The expression patterns of circRNAs in the thyroid tissues of 12 STH sheep were investigated by RNA-seq. The husbandry management and euthanasia of test animals in this study were in accordance with animal welfare guidelines.

### 2.2. RNA Extraction, Library Construction, and Sequencing

RNA was extracted from the thyroid tissue of 12 STH sheep using TRIzol reagent, and 3 μg of RNA was used for each sample. Ribosomal RNA was first removed using the Epicentre riboo-zero™ rRNA removal kit (Epicentre, Madison, WI, USA), and then the rRNA residues were removed by ethanol precipitation using the RNA Nano 6000 Assay Kit of the Bioanalyzer 2100 system (Technologies, Santa Clara, CA, USA), the NanoPhotometer^®^ spectrophotometer (IMPLEN, Westlake Village, CA, USA), and the Qubit^®^ RNA Assay Kit in the Qubit^®^ 2.0 Fluorometer (Life Technologies, San Francisco, CA, USA) to detect the purity and concentration of RNA. Sequencing libraries were constructed using the NEBNext^®^ Ultra™ Directed RNA Library Preparation Kit (NEB, Ipswich, MA USA). The steps for the library construction are as follows: (1) Fragmentation of RNA into short fragments by adding a fragmentation buffer to the system in which rRNA has been removed; (2) synthesis of the first strand of cDNA using a short fragment of RNA as a template with random primers; (3) addition of buffer, RNaseH, dNTPs, and DNA polymerase I to synthesize the second strand; (4) the product was purified using the QiaQuick PCR Kit and eluted by adding EB buffer, and the second strand was digested by adding UNG enzyme to recover the target fragment after end repair and splicing, followed by PCR amplification and recovery of the target fragment, thus completing the library construction. Finally, sequencing was performed on the Illumina Hiseq platform (Illumina, San Diego, CA, USA), and library quality was assessed on an Agilent Bioanalyzer 2100 system.

### 2.3. Sequencing Data Filtering, Comparative Analysis, and Splicing

The raw sequencing data (raw reads) were filtered to remove low-quality reads to obtain high-quality reads (clean reads), the Q30 and GC content of high-quality reads were calculated, and subsequent analyses were performed using clean reads. The genome version was Oar v. 4.0. clean reads and calculated gene expression levels were assembled using StringTie v. 1.3.1 aligned to the sheep reference genome.

### 2.4. circRNA Identification

The circRNAs studied so far are mainly exon-cyclized RNAs, which are formed by lasso-driven cyclization and intron pairing-driven cyclization. Regardless of the way the circRNA is formed, it will be formed by the splice donor (SD) of the downstream exon of the circRNA and the splice acceptor (SA) of the upstream exon after reverse splicing, so the principle of recognizing the circRNA is the sequence splitting comparison and searching for the GT-AG signal next to the junction site. Firstly, the sequences were split and compared using CIRI software (V.2.0.3), and then the SAM files in the result were scanned for PCC (paired chiastic clipping) and PEM (paired-end mapping) sites, as well as GT-AG splicing signals, and finally, the sequences with junction sites were re-compared using dynamic planning algorithm to ensure the reliability of circRNA identification. The identified circRNAs were annotated in circBase, and the circRNAs that could not be annotated were defined as NOVEL circRNAs.

### 2.5. Differential Expression Analysis

The expression levels of circRNAs were normalized using SRPBM (Spliced Reads per Billion Mapping), which eliminates the effects of sequencing depth, gene length, and inter-sample variation on gene expression and quantifies the expression of circRNAs. This experiment has three biological replicates, and the obtained circRNAs were analyzed for differential expression between groups using Deseq2 (V.1.8.1). In this study, |log2(fold change)| > 2 and *p* < 0.05 were used as the conditions for multiple differentially expressed circRNA screening.

### 2.6. Enrichment Analysis of the Source Genes of circRNAs 

GO (Gene Ontology) and KEGG (Kyoto Encyclopedia of Genes and Genomes) enrichment analyses of the target genes of differentially expressed circRNAs were performed using the DAVID online website (https://david.ncifcrf.gov, accessed on 25 January 2023). The GO analysis mainly includes biological processes (BP), cellular component (CC) and molecular function (MF) to understand the function of differentially expressed circRNAs. *p* < 0.05 was considered a significant enrichment in this pathway.

Construction of the circRNA–miRNA co-expression network

The circRNA–miRNA co-expression network was realized by Cytoscape (V3.9.1) software visualization. The data sources for the DEMs in this study are the same as the results of the previous studies [17].

### 2.7. Validation of Sequencing Data

(1) Extraction and identification of total RNA

The steps in this section are the same as in Section 2.2.

(2) Reverse transcription

The steps in this section are the same as in Section 2.2, using the reverse transcription system as shown in Table 1.

The reverse transcription system was configured as a mixed solution and placed in a PCR instrument under the following reaction conditions: 37 °C, 15 min; 85 °C, 5 s. The cDNA products obtained after reverse transcription were diluted 5 times and stored at −20 °C.

(3) Primer design and synthesis

A total of 12 differentially expressed circRNAs were selected for RT-qPCR validation, and fluorescent quantification primers were designed using the software Primer Premier 6.25. GRPDH was the internal reference gene of the circRNA, and the information on the fluorescent quantification primers is shown in Table 2.

### 2.8. Validation of circRNA–miRNA–mRNA ceRNA Mechanism

(1) Carrier construction

PCR primers were designed based on the sequence matching miR-133 in the *TXLNA* -3’UTR sequence in the NCBI database. The enzyme cut site CTCGAG of *Xho* I was added upstream of the PCR primer, and the enzyme cut site GCGGCCGC of *Not* I was added downstream. The vector used was psiCHECK2, and the PCR product was linked to the double-cleaved psiCHECK2 vector and transformed into *E. coli.* Twelve colonies of overnight cultured *E. coli* were picked separately for PCR identification, and the final *TXLNA*-3’UTR-wild-type vector (*TXLNA*-3’UTR-WT) was obtained. The *TXLNA*-3’UTR-WT vector was mutated using the site-directed mutation method, *E. coli* was transformed in the same way as described above, and the final *TXLNA*-3’UTR-mutant vector (*TXLNA*-3’UTR-MT) was obtained. *FGFR1*-3’UTR wild-type vector (*FGFR1*-3’UTR-WT) and mutant vector (*FGFR1*-3’UTR-MT) were described above.

The PCR primers were designed according to the full-length sequence of circ_0012128 in the sequencing data by adding the enzymatic cut site GGTACC of *Knp* I upstream and CTCGAG of *Xho* I downstream of the PCR primers, using the vector pcDNA3.1. Twelve colonies of E. coli were picked for PCR identification to obtain the circ_0012128 overexpression vector (pcDNA3.1-circ_0012128). circ_0003259 overexpression vector (pcDNA3.1-circ_0003259) was constructed as above.

(2) Cell culture and transfection

HEK293T cell lines were inoculated into 12-well cell culture plates containing complete medium (DMEM + 10% FBS + 1.5% double antibody) and incubated in a 37 °C cell culture incubator. Transfection was performed when the cell density reached 70–80%, and the experiment was divided into six groups, with three replicates set in each group. Group I: psiCHECK2-*TLXNA*-WT+ oar-miR-133 mimics. Group II: psiCHECK2-*TLXNA*-WT + oar-miR-133 mimics NC (oar-miR-133 mimics negative control). Group III: psiCHECK2- *TLXNA*-MT + oar-miR-133 mimics. Group IV: psiCHECK2-*TLXNA*-MT + oar-miR-133 mimics NC. Group V: psiCHECK2-*TLXNA*-WT + oar-miR-133 mimics + pcDNA3.1_circ_0003259. Group VI: psiCHECK2-*TLXNA*-WT + oar-miR-133 mimics + 2× pcDNA3.1_circ_0003259. circ_0012128-oar-miR-370-3p-*FGFR1.* The experimental design was as above.

After transfection, the cells were placed in a cell culture incubator and starved with OPTI-MEM for 6 h. The complete medium was replaced, and the culture was continued for 48 h. DMEM and OPTI-MEM were purchased from Beijing Xinhuitian Orient Technology Co., Ltd. (Beijing, China); trypsin, fetal bovine serum, agarose, tryptone, yeast extract, NaCl, kanamycin, and the QIAGEN Plasmid Midi Kit were purchased from Beijing Cui Feng Technology Co. (Beijing, China).

(3) Analysis of dual luciferase activity

After transfection for 48 h, the cells were placed on an ultra-clean table, the supernatant was aspirated, 100 μL of trypsin was added to each well, and the digestion was terminated by adding an appropriate amount of serum after 3 min. The double-luciferase activity assay was performed using the TransDetect Double-Luciferase Reporter Assay Kit. Seventy-five microliters of Luciferase Reaction Reagent was added to the cell precipitate according to the instructions, mixed thoroughly, and then the fluorescence activity was detected using a Tecan Infinite 200 Pro multifunctional enzyme marker. An additional 75 μL of Luciferase Reaction Reagent II was added at the end of the assay was completed, and 20–30 min elapsed before fluorescence activity was detected.

(4) Statistical analysis

The relative expression of circRNA was analyzed by an independent sample t-test. The dual luciferase reporter gene data were calculated from the relative luciferase activity data by a one-way ANOVA with relative luciferase activity = activity of the sea kidney luciferase reporter gene/activity of the firefly luciferase reporter gene. *p* < 0.05 indicates a significant difference. Statistical analysis and plots were done using GraphPad Prism 8.3.0.

## 3. Results

### 3.1. circRNA Characterization

**Figure 1:**
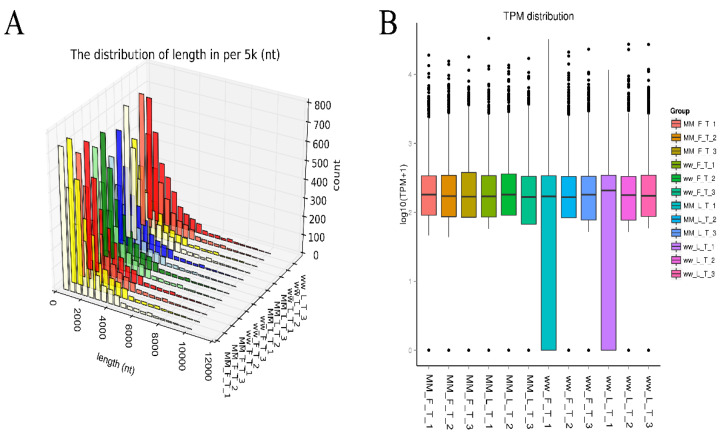
Characterization of thyroid circRNA in sheep. Characterization of thyroid circRNA in sheep. (**A**) circRNA length distribution map, (**B**) circRNA expression box diagram.

A total of 27583 circRNAs were identified from follicular and luteal phase *FecB BB* and *FecB ^++^* type STH sheep thyroid tissues according to circRNA recognition conditions (Figure 1A), with lengths mostly within 2000 nt (Figure 1B), and box plots showing that lg (TPM + 1) values were mostly between 2 and 3 (Supplementary Tables S1 and S2).

### 3.2. Differential Expression and Analysis of circRNA

**Figure 2:**
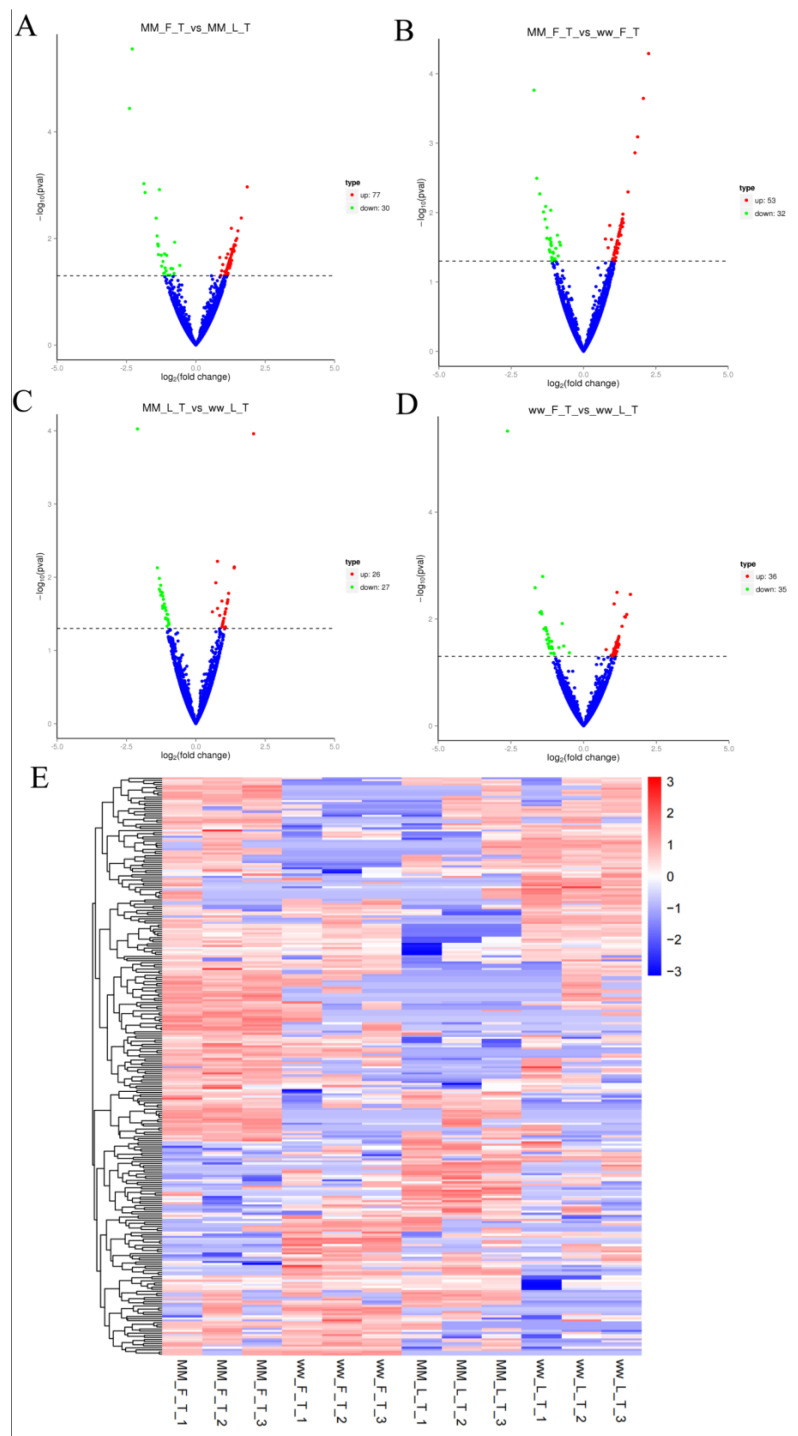
DECs analysis among 4 groups. Volcano plots show the up- and down-regulation distribution of DELs in MM-FT vs. MM-LT (**A**), MM-FT vs. ww-FT (**B**), MM-LT vs. ww-LT (**C**), and ww-FT vs. ww-LT (**D**), where red and green represent up- or down-regulation, respectively. Heatmap (**E**) shows the expression patterns of the 4 groups of DECs.

There were 107 DECs in the MM-FT vs. MM-LT group (77 up-regulated, 30 down-regulated, Figure 2A), 85 DECs in the MM-FT vs. ww-FT group (53 up-regulated, 32 down-regulated, Figure 2B), 53 DECs in the MM-LT vs. ww-LT group (26 up-regulated, 27 down-regulated, Figure 2C), and ww-FT vs. 71 DECs in the ww-LT group (36 up-regulated, 35 down-regulated, Figure 2D). Volcano and heat maps showed the number and expression pattern of DECs (Figure 2E), and all DECs (Supplementary Table S3) were statistically significant (*p* < 0.05).

### 3.3. Functional Enrichment Analysis of DECs-Derived Genes

**Figure 3:**
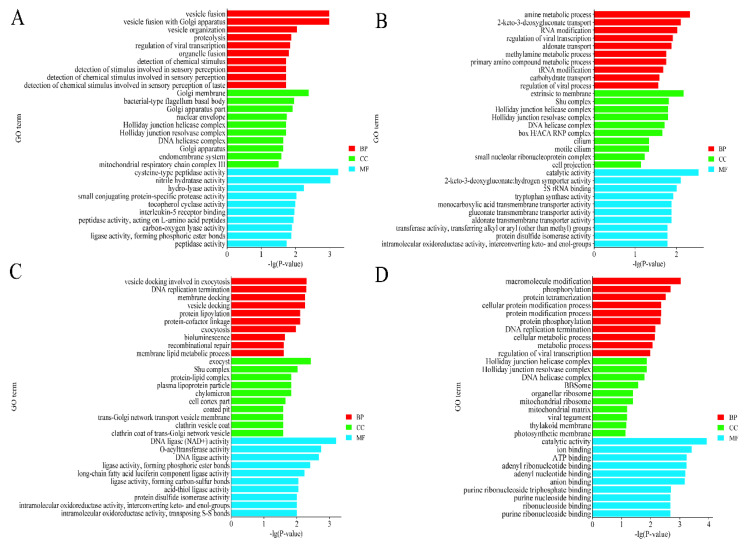
Top 10 enriched GO terms of host genes of DECs in 4 groups. The top 10 enriched GO terms of host genes of DECs in (**A**) MM-FT vs. MM-LT (**B**) MM-FT vs. ww-FT, (**C**) MM-LT vs. ww-LT, (**D**) ww-FT vs. ww-LT. The horizontal and vertical coordinates represent the GO terms and -lg(*p*-value) of the enriched genes, respectively.

GO functional annotation revealed the highest enrichment of GO entries in the three processes BP, CC, and MF for the source genes of the four groups of DECs, respectively: Vesicle fusion, Golgi membrane, and cysteine-type peptidase activity. Amine metabolic process, extrinsic to the membrane, catalytic activity. Vesicle docking is involved in exocytosis, exocyst, and DNA ligase activity. Macromolecule modification, Holliday junction helicase complex, catalytic activity. In addition, these DECs are significantly enriched in the endometrial system, RNA modification, and DNA replication termination (Figure 3).

**Figure 4:**
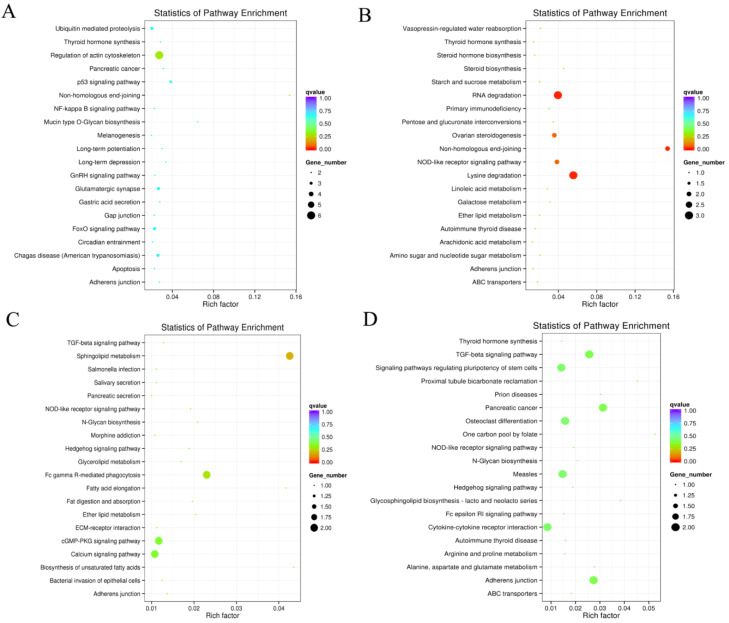
Twenty enriched KEGGs of host genes of DECs target genes in 4 groups. Twenty enriched KEGG of host genes of DECs in (**A**) MM-FT vs. MM-LT, (**B**) MM-FT vs. ww-FT, (**C**) MM-LT vs. ww-LT, (**D**) ww-FT vs. ww-LT. horizontal and vertical coordinates represent the -lg(*p*-value) of enriched genes and KEGG pathway, respectively. The rich factor is the ratio of the number of differentially expressed genes to the number of annotated genes enriched in that pathway term. The size of the circles in the graph indicates the number of differential genes enriched in the pathway.

KEGG enrichment analysis revealed that the most significant signaling pathways in the four groups in which genes of DECs origin could be significantly enriched were non-homologous end-joining, lysine degradation, sphingolipid metabolism, and pancreatic cancer. In addition, it can also be enriched in P53 signaling pathway, thyroid hormone synthesis, GnRH signaling pathway, ovarian steroidogenesis, TGF-β signaling pathway, and NF-κB signaling pathway in reproduction-related signaling pathways (Figure 4 and Supplementary Tables S4 and S5).

### 3.4. Analysis of circRNA–miRNA Co-Expression Network

**Figure 5:**
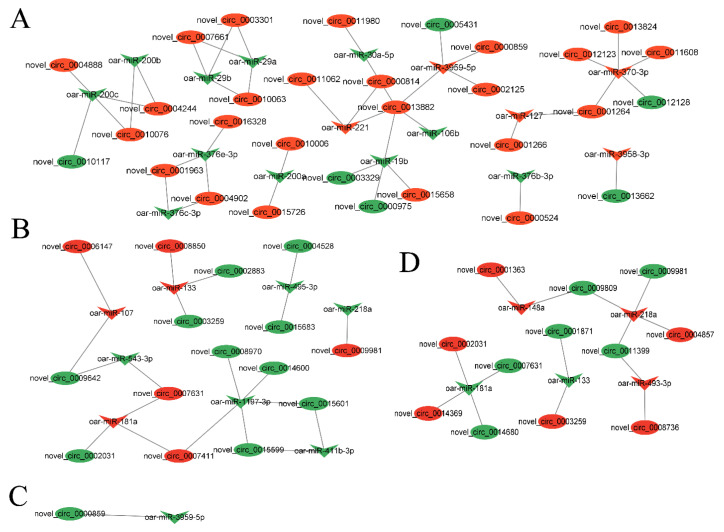
circRNA–miRNA network interaction analysis. The DECs–DEMs network between (**A**) MM-FT vs. MM-LT, (**B**) MM-FT vs. ww-FT, (**C**) MM-LT vs. ww-LT, (**D**) ww-FT vs. ww-LT. Note: Nodes represent circRNA and miRNA connecting lines represent the interaction between circRNA and miRNA, red represents circRNA, and green represents miRNA. Octagon, inverted diamond, and circle represent circRNA and miRNA, respectively.

In order to better understand the potential functionality of these DECs, we selected DECs and DEMs in each group to construct a circRNA–miRNA co-expression network. In four groups, there were 54 DECs targeted by 27 DEMs in the circRNA–miRNA co-expression network. Among them, oar-miR-370-3p and oar-miR-1197-3p target the most regulated circRNAs. Next, oar-miR-133, oar-miR-200c, oar-miR-181a, oar-miR-218a, oar-miR-3959-3p, and oar-miR-19b targeted four circRNAs (Figure 5 and Supplementary Table S6).

### 3.5. Validation of Sequencing Data

**Figure 6:**
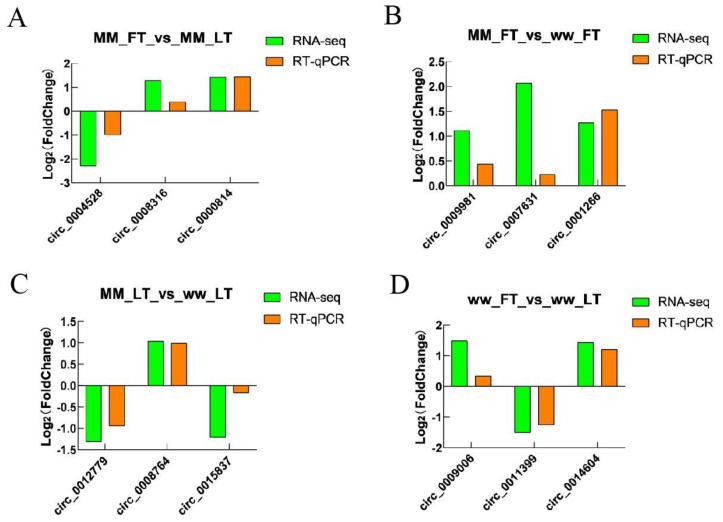
RT−qPCR verification of DECs. RT-qPCR verified the expression trend of DECs in (**A**) MM-FT vs. MM-LT, (**B**) MM-FT vs. ww-FT, (**C**) MM-LT vs. ww-LT, (**D**) ww-FT vs. ww-LT.

To verify the accuracy of the sequencing data, we randomly selected 12 circRNAs: circ_0004528, circ_0008316, circ_0000814, circ_0001266, circ_0008316, circ_0009981, circ_0007631, circ_0014604, circ_0012779, circ_0008764, circ_0015837, circ_0009509, circ_11399, and circ_0009006 were validated by RT-qPCR assay, and GRPDH was the internal reference gene. The results showed that the RT-qPCR trends and RNA-seq expression trends were consistent, indicating that the sequencing results were reliable (Figure 6; Supplementary Table S7).

### 3.6. Plasmid Construction

**Figure 7:**
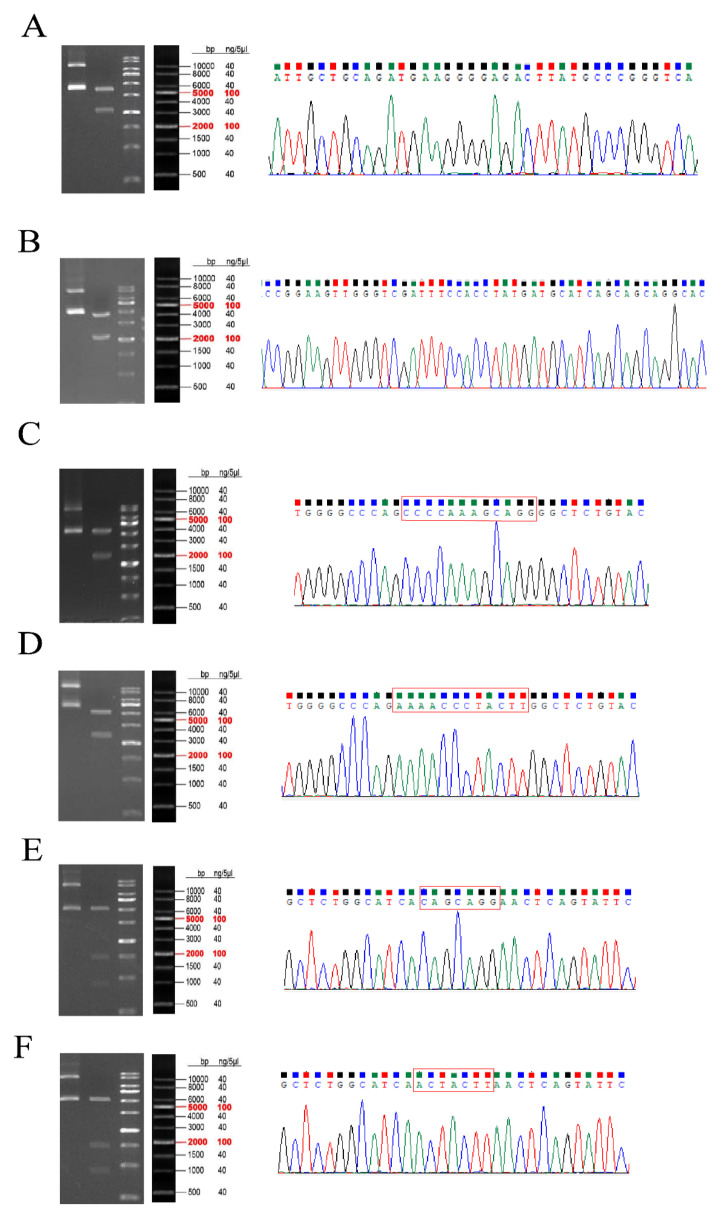
Plasmid construction results. (**A**) circ_0003259 overexpression vector. (**B**) circ_0012128 sequencing results. (**C**) *TXLNA*-3′UTR WT sequencing results. (**D**) *TXLNA*-3′UTR MT sequencing results. (**E**) *FGFR1*-3′UTR WT sequencing results. (**F**) *FGFR1*-3′UTR MT sequencing results.

The sequencing results of the circ_0003259 overexpression vector (Figure 7A) and circ_0012128 overexpression vector (Figure 7B) are shown in the figure, and the mutant plasmid sequences were the mutation of CAGCAGG to ACTACTT in *TXLNA*-3′UTR (Figure 7C,D) and the mutation of CCCCAAAGCAGG to AAAACCCTACTT in *FGFR1*-3′UTR (Figure 7E,F), which were consistent with the expected results and could be tested in the follow-up (Supplementary Table S8).

### 3.7. Experimental Validation of Dual Luciferase

**Figure 8:**
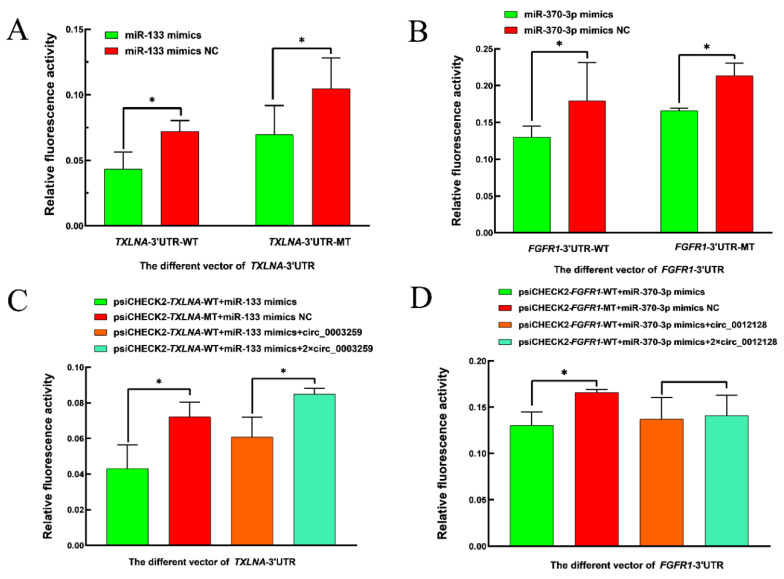
Double luciferase activity detection results. (**A**) Relative fluorescence activity was detected after cotransfection of oar-miR-133, oar-miR-133 mimics NC, *TXLNA*-WT, and *TXLNA*-MT into 293T cells. (**B**) Relative fluorescence activity was detected after cotransfection of oar-miR-370-3p mimics, oar-miR-370-3p mimics NC, *FGFR1*-WT, and *FGFR1*-MT into 293T cells. (**C**) Relative fluorescence activity was detected after cotransfection of circ_0003259, oar-miR-133 mimics, and *TXLNA* into 293T cells. (**D**) Relative fluorescence activity was detected after cotransfection of circ_0012128, oar-miR-370-3p mimics, and *FGFR1* into 293T cells. Note: “*” indicates significant differences (*p* > 0.05).

The results of the dual luciferase reporter test showed that the relative fluorescence activity of the first group in Figure 8A,B was significantly lower than the other three groups (*p* < 0.05), suggesting a possible targeting relationship between oar-miR-370-3p and *FGFR1*-3′UTR, oar-miR-133 and *TXLNA*-3′UTR, in Figure 8C, the first group had the lowest relative fluorescence activity, the third group had significantly higher relative fluorescence activity than the first group, while significantly lower than the fourth group, in Figure 8D, the first group had the lowest relative fluorescence activity, the third and fourth groups had higher relative fluorescence activity than the first group but the difference was not significant, indicating that the ceRNA mechanism between oar-miR-133 and *TXLNA* and circ_0003259, oar-miR-370-3p and *FGFR1* and circ_0012128 might hold (Supplementary Table S9).

## 4. Discussion

The *FecB* gene, also known as the *BMPR1B* gene, is the main effective gene affecting lambing number in STH sheep. It was found that the A746G mutation in the coding region of the *BMPR1B* gene resulted in the replacement of glutamine at position 249 in the protein sequence with arginine (Q249R) and that this mutation resulted in multiple lambs in sheep. Three genotypes of *FecB* were found to exist: *FecB BB*, *FecB B+*, and *FecB ++*, with *FecB B+* genotyped ewes producing one more lamb per litter than *FecB ++* genotyped ewes and *FecB BB* genotyped ewes producing 1.5 more lambs per litter than *FecB ++* genotyped ewes, and all three genotypes were distributed in STH sheep [18].

During the passage from the follicular to the luteal phase, the levels of hormones such as FSH, LH, E2, and P4 change in sheep, which in turn affects the reproductive process in sheep [19], in which FSH and LH play an important role in the development process of the ovary in sheep. FSH can maintain the number of follicles to promote follicular growth; if the lack of FSH leads to sheep primordial follicle growth and development being affected, LH is necessary to promote the development of mature follicles in sheep for the luteal body [20].

In the present study, we identified the expression profiles of circRNAs in the thyroid tissues of STH sheep with follicular and luteal phase *FecB BB* genotypes and *FecB* ++ genotypes, respectively. One hundred and seven DECs were identified in the MM-FT vs. MM-LT group. Eighty-five DECs were identified in the MM-FT vs. ww-FT group. Fifty-three DECs were identified in the MM-LT vs. ww-LT group, and 71 DECs were identified in the ww-FT vs. ww-LT group. Among the source genes of DECs, we found that the source gene of novel_circ_0003219 is guanine nucleotide-binding protein (*GNAQ*), the source gene for novel_circ_0015837 is recombinant vascular endothelial growth factor C (*VEGFC*), the source gene of novel_circ_0013082 is mitogen-activated protein kinase 1 (*MAPK1*), signal transducer and activator of transcription 1 (*STAT1*), the source gene of novel_circ_0002547, and *HSD17B7*, the source gene of novel_circ_0000106. We hypothesize that these differentially expressed circRNAs may have an important role in reproduction in sheep.

Guanine nucleotide-binding protein (GNAQ) is a member of the Gq-like subfamily of the G protein alpha-subunit multigene family and is expressed in multiple tissues, including the liver, heart, muscle, spleen, adipose tissue, brain, and uterus [21]. *GNAQ* gene polymorphisms were found to be significantly associated with lambing traits in Kazakh, Chinese Merino, and Lake sheep [22]. In the hypothalamic neuronal cells of Kazakh sheep, *GNAQ* regulates its downstream gene *PLCB1,* which directly regulates the expression and secretion of GnRH through the calcium and PRKC signaling pathways, and indirectly regulates the expression of kisspeptin through the kisspeptin-GPR54 signaling pathway, which in turn regulates the secretion of GnRH in Kazakh sheep [23]. Yurchenko et al. [24] performed high-density genotyping of the genome and a comprehensive scan of 15 Russian sheep breeds and identified *GNAQ* as an important gene affecting sheep reproduction. In conclusion, *GNAQ* in the sheep thyroid may affect the reproductive process in sheep by influencing the secretion of GnRH. 

Recombinant vascular endothelial growth factor C (VEGFC) acts as a transcription factor that controls the expression of a large number of genes that play important roles in mammalian metabolism, fertility, immunity, angiogenesis, follicular growth, and ovulation [25]. Hayashi et al. [26] identified the endometrial *VEGFC* mRNA expression pattern and protein localization in cattle during different estrus periods and found that *VEGFC* regulates uterine function in the peri-uterine phase. 

Mitogen-activated protein kinase 1 (MAPK1) is thought to be a regulator of oocyte survival [27]. Activation of MAPK1 increases cortical granule migration and mitochondrial activity in bovine oocytes and improves oocyte nuclear and cytoplasmic maturation, which in turn affects embryo development and quality [28]. Inhibition of MAPK1 inhibits angiogenesis during the conversion of bovine follicles to the corpus luteum [29].

Signal transducer and activator of transcription 1 (*STAT1*) is a gene associated with the ability of blastocyst development in the study of in vitro bovine embryo morphodynamics [30]. Heat stress affects the estrous cycle by affecting the ovarian JAK/STAT1 signaling pathway, which in turn leads to infertility in pigs [31]. Liu et al. [32] performed RNA-seq analysis of goat ovaries in the follicular and luteal phases and found *HSD17B7* to be a differentially expressed gene, implying that *HSD17B7* is a gene that regulates ovarian development in goats. *HSD17B7* is also involved in regulating the steroid hormone synthesis pathway and the differentiation of chicken embryonic stem cells into spermatogonial stem cells [33].

GO analysis revealed that these differentially expressed circRNA-derived genes can be enriched in pathways such as the endometrial system, vesicle fusion, protein hydrolysis, RNA modification, DNA replication termination, DNA resolvase complexes, and chromatin binding, which are widely involved in biological processes such as metabolism and reproduction in animals.

KEGG analysis revealed that these genes could be enriched in the TGF-β signaling pathway, p53 signaling pathway, GnRH signaling pathway, NF-κB signaling pathway, ovarian steroidogenesis, and other signaling pathways related to the animal reproductive process. The TGF-β signaling pathway is important in mammals, and studies have shown that TGF-β1 mediates *MMP1* down-regulation of type I collagen deposition in granulosa cells through the AKT/GSK-3β signaling pathway, which inhibits luteinization and progesterone production in granulosa cells [34]. It also down-regulates *DHCR24* expression via the GSK-3β/EZH2/H3K27me3 signaling pathway, which in turn inhibits cholesterol denovo synthesis in granulosa-lutein cells [35]. TGF-β1/SMAD4/miR-183-96-182/FoxO1 may be one of the pathways regulating follicular atresia and female reproduction [36]. P53-Sox3 signaling regulates spermatogenesis and male fertility [37]. The FOXO1-TP53INP1 axis inhibits the G0/G1 cycle of human granulosa cells through the p53-CDKN1A signaling pathway, which in turn leads to abnormal follicle development [38]. Treatment of cultured chicken ovarian cells in vitro with melatonin reveals that melatonin ameliorates ovarian oxidative stress via the SIRT1-P53/FoxO1 pathway [39]. Treatment of cultured chicken ovarian cells in vitro with melatonin reveals that melatonin ameliorates ovarian oxidative stress via the SIRT1-P53/FoxO1 pathway [40,41]. The NF-κB signaling pathway is a key pathway that regulates cellular redox status and anti-stress and anti-inflammatory responses [42], miR-93-5p promotes apoptosis of granulosa cells in polycystic ovary syndrome through the NF-κB signaling pathway [43], and *GNRB1* inhibits lipopolysaccharide-induced endometritis in mice by suppressing the NF-κB signaling pathway [44]. In addition, there are signaling pathways such as ovarian steroid synthesis [45] and thyroid hormone synthesis [46] that also play important roles in animal reproduction. 

To better understand the mechanism of the role of DECs in sheep reproduction, we constructed a circRNA–miRNA co-expression network in which there are multiple pairs of circRNA–miRNA regulatory relationships, and there are also multiple DEGs with genes originating from DECs involved in the reproduction process of the animals, suggesting that these DECs play an important role in the reproduction process of sheep. In the circRNA–miRNA co-expression network, oar-miR-370-3p and oar-miR-133 could target the most DECs, suggesting that oar-miR-370-3p and oar-miR-133 are located in the core of the co-expression network, and their targeting of DECs may influence the reproductive process of sheep by affecting the sheep thyroid gland. Subsequently, we performed differentially expressed circ_0003259 and circ_0012128 for ceRNA mechanism validation. The source genes of circ_0003259 and circ_0012128 are two genes, *TRPM3* and *RIC3*, respectively, both of which may be involved in the reproductive process of animals [47,48]. *FGFR1* is a tyrosine kinase receptor that can regulate embryonic development by binding to fibroblast growth factor and then activating a series of downstream pathways [49]. The *FGFR1* gene also regulates the migration of gonadotropic GnRH neurons, which in turn are involved in the reproductive process of animals [50]. Liu et al. [51] performed RNA-seq analysis on the ovarian tissues of goats with high and low follicular fertility and identified several miRNAs and mRNAs, and *TXLNA* was found to be the gene affecting fertility in sheep. Currently, there is no report that *TXLNA* and *FGFR1* genes can affect the reproductive process of animals through the thyroid gland, and the specific mechanism of circ_0003259-oar-miR-133-*TXLNA* and circ_0012128-oar-miR-452 370-3p-*FGFR1* genes affecting the reproductive process of sheep through the thyroid gland needs to be further explored.

## 5. Conclusions

Through RNA-seq analysis of thyroid tissues from STH sheep, we found that multiple DECs were present in each of the four groups: MM-FT vs. MM-LT, MM-FT vs. ww-FT, MM-LT vs. ww-LT, and ww-FT vs. ww-LT. The GO enrichment results revealed that MM-FT vs. MM-LT was enriched to the most significant GO terms of vesicle fusion, Golgi membrane, cysteine-type peptidase activity, MM-FT vs. ww-FT was enriched to the most significant GO terms of amine metabolic process, extrinsic to the membrane, catalytic activity, MM-LT vs. ww-LT was enriched to the most significant GO terms of vesicle docking involved in exocytosis, exocyst, DNA ligase activity, and ww-FT vs. ww-LT was enriched to the most significant GO terms were macromolecule modification, Holliday junction helicase complex, and catalytic activity. KEGG enrichment analysis revealed that these DECs-derived genes were significantly enriched in TGF-β signaling pathway, P53 signaling pathway, GnRH signaling pathway, NF-κB signaling pathway, and ovarian steroid synthesis involved in the animal reproductive process. The target gene prediction results showed that a total of 54 DECs interacted with 27 DEMs in four groups. Among them, oar-miR-370-3p targeted the most regulated circRNAs, targeting five circRNAs, followed by oar-miR-133 targeting three circRNAs in the MM-FT vs. ww-FT group. circ_0003259-oar-miR-133-*TXLNA* and circ_0012128-oar-miR-370-3p-*FGFR1* may have ceRNA regulatory mechanisms that warrant further study in the future to provide insight into the mechanism of high reproduction in sheep.

## Figures and Tables

**Table 1:** The amount of reverse transcription reagents used.

Reagents	Volume
PrimeScript RT Enzyme Mix I	1 µL
Oligo dT Primer	1 µL
Random 6 Mers	1 µL
5 × PrimeScript Buffer	4 µL
Total RNA (500 ng/µL)	2 µL
RNase-Free ddH2O	11 µL

**Table 2:** The primer sequences designed for real-time fluorescence quantification of circRNA.

Gene Name	Primer Sequences (5’-3’)	Accession No.	Tm (°C)
circ_0004528	F: TGATGAGCAGCTTTGCAGAAGA	101123020	60
	R: CTCGTGCATGCGTCTCTTGAC		
circ_0008316	F: AGCCAACAAGATGAGATCGACAG	101103576	60
	R: CTCAAGTTCCTCGTGCTGGGA		
circ_0000814	F: AGGGGAAAAGTCCTTGATGCAA	101106049	60
	R: GCTACATCCATCTTTGAACGTGC		
circ_0009981	F: GGGATCAAGGTCATTCACAAGC	101102742	60
	R: TTTCTCCCCAGCCAGTACAAAG		
circ_0007631	F: GGCTGTCTCTATTTTGGGAGATG	101112983	60
	R: GCATAGGCCTTTCCTTCTGTG		
circ_0001266	F: TCAGCTACACAGTTGCCCCAA	101115254	60
	R: TCTTTCCCCTGAGTTGCCTCG		
circ_0012779	F: GCAACTGCTACTGGAAGAGGAG	101101794	60
	R: ACTGGCTCATGCTTCACTGGA		
circ_0008764	F: CTGGTCTCTGGAGGTGTTCTG	101105760	60
	R: CGTTGCTGCAAGTTCACTCC		
circ_0015837	F: TGTGTCCGTCTACAGATGTGG	101105629	60
	R: GTCATGAGTTCATCCACACTGG		
circ_0009006	F: GTCCTGGACTTGGCCGTGTAT	101102570	60
	R: CCGGCACCACTTGTCAATGTG		
circ_0011399	F: CAGTTCCCTGGGTTTGCACAC	101121697	60
	R: GGGACACACACTGAACCAGCT		
circ_0014604	F: ACGTGAACCTTGCTTCTGGC	101119353	60
	R: TGCGATGGAGCAGAGCAGTT		
GRPDH	F: ATCGCCAATGCCAACTC	NM_001190390.1	60
	R: CCTTTCGCTTACCTATACC		

## Data Availability

The original contributions presented in the study are included in the article and supplementary material, further inquiries can be directed to the corresponding authors.

## Supplementary Materials

The following supporting information can be downloaded at: https://www.mdpi.com/article/10.3390/ani14010105/s1. Supplementary Table S1: Length of total circRNAs in four groups. Supplementary Table S2: Expression of total circRNAs in four groups. Supplementary Table S3: Total set of DECs was up-and-down-regulated in four groups. Supplementary Table S4: GO enrichment of differentially expressed DECs targets in four groups. Supplementary Table S5: Top 20 KEGG enrichment pathways for differentially expressed DECs targets in four groups. Supplementary Table S6: Co-expression details of circRNA–miRNA network in four groups. Supplementary Table S7: QPCR data of DECs in four groups. Supplementary Table S8: Sequence of the constructed vector. Supplementary Table S9: Dual luciferase report experimental data.
