# Supplementary material for: Transcriptome Analysis Reveals Differentially Expressed circRNAs Associated with Fecundity in Small-Tail Han Sheep Thyroid with Different FecB Genotypes
Source: Animals (Basel). 2023 Dec 27;14(1):105. doi: 10.3390/ani14010105 (PMC10777913; doi:10.3390/ani14010105)
Supplement: Supplementary file 1 [file animals-14-00105-s001.zip › Description of supplementary materials/Description of supplementary materials.docx]

Supplementary Table S1: Length of total circRNAs in four groups.

Supplementary Table S2: Expression of total circRNAs in four groups.

Supplementary Table S3: Total set of DECs was up-and-down-regulated in four groups.

Supplementary Table S4: GO enrichment of differentially expressed DECs targets in four groups.

Supplementary Table S5: Top 20 KEGG enrichment pathways for differentially expressed DECs targets in four groups.

Supplementary Table S6: Co-expression details of circRNA-miRNA-mRNA network in four groups.

Supplementary Table S7: QPCR data of DECs in four groups.

Supplementary Table S8: Sequence of the constructed vector.

Supplementary Table S9: Dual luciferase report experimental data.
